# BMPR2 expression is suppressed by signaling through the estrogen receptor

**DOI:** 10.1186/2042-6410-3-6

**Published:** 2012-02-20

**Authors:** Eric D Austin, Rizwan Hamid, Anna R Hemnes, James E Loyd, Tom Blackwell, Chang Yu, John A Phillips III, Radhika Gaddipati, Santhi Gladson, Everett Gu, James West, Kirk B Lane

**Affiliations:** 1 Department of Pediatrics, Vanderbilt University Medical Center, 1161 21st Ave S Suite DD-2205, Nashville, TN 37232-2578, USA; 2 Department of Medicine, Vanderbilt University Medical Center, 1161 21st Ave S Suite DD-2205, Nashville, TN 37232-2578, USA; 3 Department of Biostatistics, Vanderbilt University Medical Center, 1161 21st Ave S Suite DD-2205, Nashville, TN 37232-2578, USA

**Keywords:** BMPR2, estrogen, hormones, expression, pulmonary hypertension

## Abstract

**Background:**

Studies in multiple organ systems have shown cross-talk between signaling through the bone morphogenetic protein receptor type 2 (BMPR2) and estrogen pathways. In humans, pulmonary arterial hypertension (PAH) has a female predominance, and is associated with decreased BMPR2 expression. The goal of this study was to determine if estrogens suppress BMPR2 expression.

**Methods:**

A variety of techniques were utilized across several model platforms to evaluate the relationship between estrogens and BMPR2 gene expression. We used quantitative RT-PCR, gel mobility shift, and luciferase activity assays in human samples, live mice, and cell culture.

**Results:**

BMPR2 expression is reduced in lymphocytes from female patients compared with male patients, and in whole lungs from female mice compared with male mice. There is an evolutionarily conserved estrogen receptor binding site in the BMPR2 promoter, which binds estrogen receptor by gel-shift assay. Increased exogenous estrogen decreases BMPR2 expression in cell culture, particularly when induced to proliferate. Transfection of increasing quantities of estrogen receptor alpha correlates strongly with decreasing expression of BMPR2.

**Conclusions:**

BMPR2 gene expression is reduced in females compared to males in live humans and in mice, likely through direct estrogen receptor alpha binding to the BMPR2 promoter. This reduced BMPR2 expression may contribute to the increased prevalence of PAH in females.

## Background

Bone morphogenetic protein receptor type 2 (BMPR2) is essential for development; mice lacking the gene fail to progress to gastrulation [1]. BMP signaling is essential in almost all tubular organogenesis. In lung, kidney and lacrimal gland formation BMP signaling is central to development [2-4]. The BMP pathway also plays a central role in Müllerian duct regression and Sertoli cell maturation regression [5,6].

Beyond its developmental role, signaling through BMPR2 is associated with several adult diseases, including arthritis, pulmonary hypertension, atherosclerosis, diabetic nephropathy, renal fibrosis, and osteoporosis [7-10]. The common role for the BMP pathway in all of these seems to be an inappropriate response to damage to the respective organ. Many of these diseases show a marked gender imbalance in prevalence, and cross-talk between estrogen and BMP signaling has been noted in systems throughout the body [11-13].

For instance, in most but not all forms of pulmonary hypertension, such as Diagnostic Group 1, females have a higher prevalence of disease [14]. However, the precise magnitude of distribution by gender is of some debate in heritable and sporadic forms of pulmonary arterial hypertension (PAH). The landmark NIH Registry study in the 1980s, composed primarily of patients with sporadic PAH, reported a female: male ratio of 1.7:1 [15]. Two large recent studies have confirmed the gender predominance, although among those with heritable and sporadic PAH the magnitude of the difference varied between 1.9:1 and 4:1 [16,17]. Moreover, while the heritable form of disease is associated with BMPR2 mutation [9], even low expression of normal BMPR2 appears to predispose to disease [9,18]. We previously demonstrated that alterations in estrogen metabolism and estrogen metabolites associate with the penetrance of PAH among female patients with a BMPR2 mutation [19]. Our work to date has focused upon the estrogen component of the sex hormone pathway in PAH, although we recognize that testosterone, dehydroepiandrosterone, and other androgens may influence both BMPR2 gene expression as well as pulmonary vasodilation [20]. Together, these data led us to question whether there might be direct regulation of BMPR2 expression by estrogen. In this study, we tested the hypothesis that estrogens directly regulate BMPR2 expression using quantitative RT-PCR, gel mobility shift, and luciferase activity assays in human samples, live mice, and cell culture.

## Methods

It is known that there is a strong gender bias in the incidence and prevalence of PAH (more females), and that BMPR2 expression is reduced in the lungs of patients with heritable PAH and other forms of PAH [15-17,21]. We hypothesized that elevated exposure to estrogens explains the gender discrepancy by directly regulating BMPR2 gene expression. We employed the following methods to test the hypothesis that estrogens directly regulate BMPR2 gene expression.

### Cultured human lymphocyte specimen studies: cell culture

Cultured lymphocyte (CL) cell lines from normal subjects were obtained from the Coriell Cell Repositories (Camden, NJ, USA). All CL cell lines were grown in 15% FBS in RPMI 1640 with 2 mM L-Glutamine for studies with and without estradiol (E2, 17β-estradiol) exposure. FBS was stripped with activated charcoal (1 g per 50 ml of FBS) for 30 min at 4°C, filtered, and sterilized. E2 was purchased from Sigma Chemical Company (St Louis, MO, USA), with stock solution made in 95% ethanol. Cells were exposed to either standard or E2-containing media. For E2-containing media, E2 was added to standard media to create a media containing 1 μM E2. Cells were exposed to standard media or E2-containing media for 24 h then harvested.

### Cultured human pulmonary microvascular endothelial cell (PMVEC) specimen studies: cell culture

An established cultured human PMVEC line was obtained for study from Drs Krump-Konvalinkova and Kirkpatrick [22]. The cells were grown in normal endothelial cell growth media from PromoCell (Heidelberg, Germany) to passage 2, and transitioned to phenol red-free media upon transition to passage 3 for E2 exposure studies. The media was changed daily for at least 48 h until the cells were at approximately 70% plate confluence, at which time exposure to normal media or media treated with E2 (1 μM) was performed.

### Cultured NMuMG cells

Normal mouse mammary gland epithelial cells (NMuMG, ATCC CRL-1636 European Collection of Cell Cultures no. 94081121) constitutively competent for TGF-ß superfamily signaling were used to measure the influence of Phorbol 12-myristate 13-acetate (PMA) and E2 upon BMPR2 gene expression [23]. We chose to use this cell line because of the competence of TGF-ß signaling, and also as a system previously validated for the study of BMPR2 gene expression [24]. They were cultured in DMEM 10% FBS, 1% pen/Strep 1% glutamine. For BMPR2 gene expression studies in response to PMA (1 μM) (Sigma Chemical Company, St Louis, MO, USA) and E2 (10^-8 ^M), cell culture media was changed 2 days prior to experimental procedures to phenol-red free, 2.5% charcoal scrubbed media.

### Cultured COS-7 cells

African green monkey kidney cells (COS-7) were obtained from American Type Culture Collection (ATCC CRL-1651, Manassas, VA, USA). COS-7 cells were chosen because they do not constitutively express estrogen receptors and are not estrogen responsive. They were thus used for the luciferase assay experiments below to further define the association of the estrogen receptor activation with BMPR gene expression. COS-7 cells were maintained in DMEM supplemented with 10% FBS and 1% antibiotic-antimycotic at 37°C and 5% CO_2 _in humidified incubator. Two days prior to experimentation the media was changed to phenol-red free, 2.5% charcoal scrubbed media.

### Electrophoretic mobility shift assay (EMSA)

NMuMG cells were grown as described above. Nuclear proteins were isolated following the protocol of Deryckere *et al. *[25]. Nuclear proteins were incubated with BMPR2 promoter derived (AGTCGGGAACTAGTTCTGACCCTCGCCCCC) or vitelligenin ER binding site (AAAACGTTCGAGGAGGTCACAGTGACCTGGAGCGG) biotinylated double stranded oligos as per the manufactures instructions. (LightShift Chemiluminescent EMSA Kit Thermo Scientific, Rockford, IL, USA). Following binding, complexes were diluted in gel-loading buffer and electrophoresed. The separated material was transferred to nylon membrane and cross-linked all following the manufacture's protocol. Detection again followed the manufacture's method, in brief, the membrane was blocked, washed, and incubated with streptavidin conjugated HRP. The conjugate is incubated, washed and detected with Luminol/enhancer solution and the image capture via X-ray film.

### Cultured lymphocyte, PMVEC, and NMuMG specimen studies

#### Analysis of BMPR2 transcripts by real time

Quantitative analysis of BMPR2 transcripts by cultured lymphocytes and PMVECs was performed using real-time RT-PCR, as previously described [18]. Briefly, cDNA was synthesized from 1 μg of total cellular RNA using Superscript III cDNA Synthesis Kit (Invitrogen, Carlsbad, CA, USA). Taqman real-time assay was designed for BMPR2 transcripts (NM_001204.6) using the Primer Express software package (Applied Biosystems, Foster City, CA, USA). Sequences were chosen for forward and reverse primers (WT-forward primer 5' TTAGTGACTTTGGACTGTCCATGAG-3'; WT-reverse primer 5'-TCTAGCACTTCTGGTGCCATATATCT-3'), as well as the probe sequence WT-Taqman probe 5'-FAM-TAAGCGAGGTTGGCACT-3'. Assays were optimized using the standard curve method, and all specimens had amplification efficiencies of > 99.5%. Real-time PCR analysis was carried out using Taqman Universal Master Mix and a 7500 Real-Time PCR system according to the manufacturer's instructions (Applied Biosystems, Foster City, CA, USA). We used the TaqMan human endogenous control plate (catalog number 4309199) to analyze 13 genes as potential housekeeping genes (18S rRNA, acidic ribosomal protein, beta-actin, cyclophilin, glyceraldehyde-3-phosphate dehydrogenase, phosphoglycerokinase, β2-microglobulin, β-glucronidase, hypoxanthine ribosyl transferase [HPRT], transcription factor IID, TATA binding protein, 7 transferrin receptor, and ABL) and chose HPRT for data normalization as previously described [26]. Each measurement was made in triplicate and expressed relative to the detection of the standard HPRT. Amplification parameters consisted of initial denaturation at 95°C for 10 min followed by 40 cycles of denaturation at 95°C for 15 s and annealing and extension at 60°C for 1 min. Relative expression levels were calculated using the comparative Ct method [27,28].

Quantitative analysis of BMPR2 transcripts by NMuMG cells was performed using real-time RT-PCR, with each measurement made in triplicate and expressed relative to the detection of the standard β-actin.

#### Northern blotting

Equal amounts (15 μg) of RNA were denatured in formaldehyde, separated by 1% agarose gel electrophoresis and transferred to a nitrocellulose membrane (Hybond N^+^) (Amersham Pharmacia, Piscataway, NJ, USA), followed by ultraviolet cross-linking. ULTRAHyb hybridization solution (Ambion, Austin, TX, USA) was used according to the manufacturer's protocol. The blots were hybridized with a cDNA probe labeled with biotin. Membranes were washed in 2 × SSC for 15 min at 42°C and then in 0.1 × SSC for 30 min at 65°C. For visualization, X-ray film was exposed for 4 h to membranes developed with horseradish peroxidase-conjugated streptavidin.

### Construction of luciferase reporter plasmids

The human BMPR2 promoter was cloned by PCR amplification with restriction endonucleas recognition site-containing primers. This amplicon was appropriately digested and cloned in to pGL3E (Promega, Madison WI. USA). The construct was confirmed by sequencing.

pBRE plasmid is a luciferase reporter, containing parts of the Id1 promoter, which responds specifically to BMP activity to reflect canonical signaling [29].

### Luciferase assay

Cytomegalovirus immediate early promoter driven human ERS-1 expression plasmids (a generous gift from M Melner) at various concentrations as reported in the results section, BMPR2-luciferase reporter plasmids and pTK-renilla, at constant concentrations (9 and 1 respectively), were transfected into COS-7 cells (ATCC CRL-1651) utilizing FuGene 6 (Roche Biochemicals, Indianapolis, IN, USA) 3:1 lipid to DNA. The total DNA amount applied was held constant by the addition promoterless plasmid. Following transfection the COS-7 were incubated for 16 h and then luciferase and renilla luciferase expression determined with the dual-luciferase assay following the manufacturer's instructions (Promega, Madison, WI, USA). Assays were read on a Turner Biosystems 20/20 n Luminometer. Luciferase expression was normalized to renilla level. All conditions were assayed in triplicate and two independent experiments were conducted.

For the pBRE assay, cells were plated into six well plates and grown to 70% to 80% confluence. Wells were transfected by application of 0.9 μg of pBRE and 0.1 μg pTK-renilla complexed with 3 μg of FuGene 6 (Roche Biochemicals, Indianapolis, IN, USA) prepared following the manufactures directions. After 16 h the media was replaced with phenol red-free media. Twenty-four hours later, wells were treated with estrogenic compounds or vehicle as described. Following a 6-h exposure, the cells were prepared for the dual-luciferase assay (Promega, Madison, WI, USA) following the manufacturer's instructions. Assays were read on a Turner Biosystems 20/20 n Luminometer. All conditions were assayed in triplicate and two independent experiments were conducted. The BMP-responsive luciferase output was normalized to renilla expression to normalize for transfection efficiency variation.

### Animal studies, ovariectomy

All studies were conducted in accordance with principles and procedures outlined in the National Institutes of Health Guide for the Care and Use of Laboratory Animals, and were approved by the Vanderbilt Institutional Animal Care & Use Committee. Wild-type male and female FVB/NJ mice were purchased from Jackson labs, bred at Vanderbilt, and pups allowed to reach adulthood. Female mice aged 8 to 9 weeks were ovariectomized 5 weeks prior to study under continuous isoflurane anesthesia. All mice were otherwise treated the same.

### Animal studies, gene expression studies

Mice were sacrificed at 13 to 14 weeks of age, and RNA extracted from whole lung homogenate using RNAeasy Protect mini kit (Qiagen, Valencia, CA, USA) according the manufacturer's protocol. Primers were designed using the NCBI primer designing tool, which incorporates BLAST searches to ensure specificity [30]. The following primer sets specific for Hprt, Bmpr2, Erα, and Erβ were used: Hprt(TGCTCGAGATGTCATGAAGGAG, TTTAATGTAATCCAGCAGGTCAGC), Bmpr2(CAGCTGGCCAGGCAGCCAAC, TGGCCAGCCTGTTGCTCTCG), Erα(CTACGGCCAGTCGGGCATCG, GCATCAGCGGGCTAGGCGAC), and Erβ(CTGCCAGGCCTGCCGACTTC, ATGCACCTGCTCGCTGGCAC). The PCR reactions and relative quantifications were performed using 25 ng of cDNA per reaction in a 7300 Real-Time PCR System (Applied BioSystems, Foster City, CA, USA). Each measurement was made in triplicate and expressed relative to the detection of the standard HPRT.

### Statistical analysis

All BMPR2 gene expression laboratory experiments were performed in triplicate. Expression values are reported as the mean relative to the housekeeping gene ± standard deviation (SD). For comparisons, data are transformed so that all values in a given comparison are relative to the lower value group which is identified as the reference and thus with a value of 1.0. Raw values were tested for normal distributions. Statistical significance was evaluated using the Student's t test or Mann-Whitney U test depending on the normality of the data. All *P *values are two-sided and a *P *value < 0.05 is considered statistically significant. Statistical analysis was performed using the statistical package SPSS for Windows (Version 16.0, SPSS Inc., Chicago, IL, USA).

## Results

### Quantitative analysis of transcripts in humans and mice demonstrate reduced BMPR2 and increased ERα and ERβ gene expression among females

**Figure 1:**
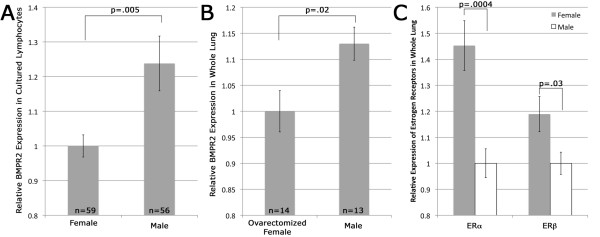
**BMPR2 expression is lower in female humans and mice than males**. **(A) **BMPR2 expression by quantitative RT-PCR, normalized to HPRT, is reduced in cultured human lymphocytes from females compared with males. **(B) **BMPR2 expression normalized to HPRT is lower in whole lung from ovariectomized female mice compared with males. **(C) **ERα and ERβ expression are both higher in whole lung from ovariectomized female mice compared with males. All comparisons were by unpaired Student's t-test.

Cultured lymphocytes provide an *in vitro *model in which to evaluate gene expression distinct from the human subject milieu; specifically, all cells are passed and exposed to equivalent levels of hormones and other factors according to the media in which they are grown. Thus, lymphocytes from 115 normal subjects were analyzed for relative BMPR2 gene expression. Of note, there was no difference between these subjects according to gender (60 females, 55 males), nor in terms of mean age (± SD) at the initial blood acquisition for the establishment of cultured cells according to gender (females 38.2 ± 24.4 years vs. males 35.1 ± 26.7 years; *P *= 0.38 Mann-Whitney U test). While there was variability among all subjects in terms of relative BMPR2 gene expression, analysis according to gender revealed a statistically significant difference between females (0.045 ± 0.011) and males (0.056 ± 0.027). Compared to females, BMPR2 gene expression was about 20% higher among males (*P *= 0.005, Figure 1A). Culture conditions were identical for all cells, so that all cells were exposed to the same level of estrogens and other hormones *in vitro*.

We next sought to confirm the differential BMPR2 gene expression between males and females *in vivo*, independent of estrogen levels. We analyzed quantitative Bmpr2 gene expression in whole lungs of ovariectomized female mice compared to male mice maintained otherwise identically, and again found a statistically significant difference in terms of Bmpr2 gene expression. Consistent with the human lymphocytes, compared to ovariectomized females, Bmpr2 gene expression was 1.13-fold higher among normal male mice (*P *= 0.020, Figure 1B).

A possible explanation for the differential BMPR2 expression between males and females in both the human cells and mouse lungs is differential expression of estrogen receptors. We therefore evaluated estrogen receptor (ER) gene expression in the same mice. Ovariectomized female mice had a relative ERα gene expression 1.45-fold higher (*P *= 0.0004), and relative ERβ gene expression 1.19-fold higher (*P *= 0.030) than male mice (Figure 1C).

### The BMPR2 promoter contains a highly conserved functional ER binding site

**Figure 2:**
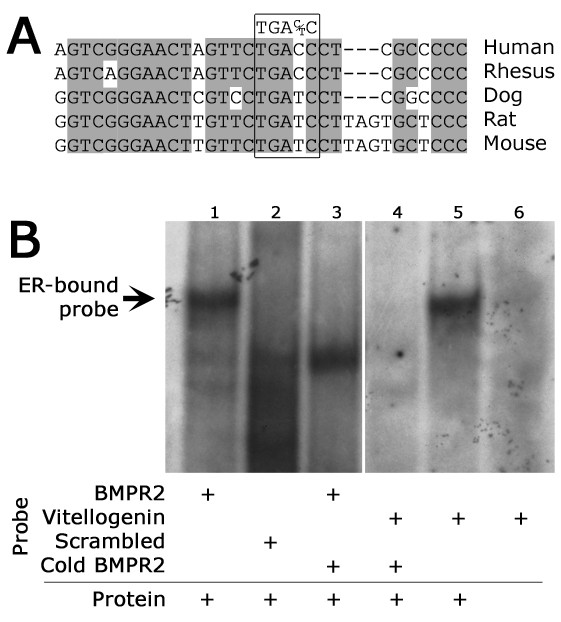
**The BMPR2 promoter contains a functional estrogen receptor binding site**. **(A) **Human BMPR2 promoter sequence (top line) containing an estrogen receptor binding site (boxed), used for gel-shift assay below, is conserved across species. **(B) **Gel-shift assay indicating that the estrogen receptor binding site is functional. BMPR2 probe sequence binds a protein (lane 1) of identical size to that bound by the canonical estrogen receptor binding site in Vitellogenin (lane 5). Binding to both the BMPR2 probe sequence and the Vitellogenin probe sequence can be competed away using cold BMPR2 probe sequence (lanes 3 and 4). Protein binding is not found when protein is omitted (lane 6) or with use of scrambled probe sequence (lane 2).

The above observations suggested that estrogen receptor binding may suppress BMPR2 expression. Examination of the BMPR2 promoter located an estrogen response element (ERE) highly conserved across species (Figure 2A). To assess the potential functionality of this site, we performed EMSAs using nuclear extracts from NMuMG cells and found that the presumed ERE within the BMPR2 promoter does bind ER *in vitro*. NMuMG cells were chosen given their prior validation in the study of BMPR2 gene expression associated with the consistent competence of their TGF-ß signaling [24]. As seen in Figure 2B, nuclear proteins were incubated with labeled oligonucleotides and competitors as indicated. The vitellogenin gene was used as a positive control, since it has a well defined ERE [31]. Exposure of the BMPR2 oligonucleotide sequence to protein results in a DNA-protein complex (lane 1) of identical size to that bound by the canonical ERE in vitellogenin (lane 5), indicating a robust binding of the ER to the BMPR2 promoter of the same mobility as the published positive control vitellogenin. The use of unlabeled ("cold") BMPR2 oligonucleotide successfully competes away this band for both the BMPR2 and the vitellogenin gene control lanes (lanes 3 and 4), which confirms the ability of the BMPR2 sequence to compete for ER binding (all experiments were done in the presence of 1,000-fold molar excess of non-specific oligonucleotide, which suggests that the elimination of vitellogenin binding is specific to the BMPR2 competition); this suggests a specificity of effect by BMPR2. The shifted band does not occur in setting of a scrambled BMPR2 oligonucleotide sequence (lane 2) or the absence of nuclear protein (lane 6), demonstrating the need for both the correct ER protein and the correct BMPR2 promoter sequence in this assay.

### Treatment of normal human cell lines with estrogens results in reduced BMPR2 gene expression, and this is amplified in the setting of cellular proliferation

**Figure 3:**
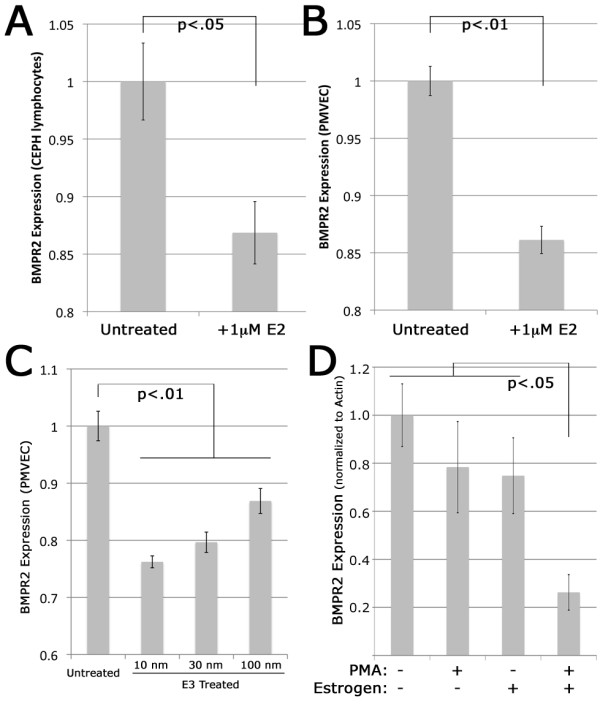
**Treatment with different estrogens reduces BMPR2 mRNA expression in multiple normal cell types**. **(A) **BMPR2 expression by quantitative RT-PCR, normalized to HPRT, is reduced in cultured human lymphocytes treated with 1 μM E2 compared to untreated controls (*P *< 0.05). **(B) **BMPR2 expression by quantitative RT-PCR, normalized to HPRT, is reduced in cultured PMVECs treated with 1 μM E2 compared to untreated controls (*P *< 0.01). **(C) **BMPR2 expression by quantitative RT-PCR, normalized to HPRT, is reduced in cultured PMVECs treated with varying doses of E3 compared to untreated controls (*P *< 0.01 for each comparison). **(D) **Estrogen and induction of proliferation through PMA both independently inhibit BMPR2 expression, by densitometry of northern blot (three replicates per condition) at *P *< 0.05 by two-way ANOVA. The combination of proliferation and estrogen produces an approximately 4× inhibition of BMPR2 expression (*P *< 0.05 by Student's t test with adjustment for multiple comparisons).

Expression of BMPR2 mRNA by both human lymphocytes and human pulmonary microvascular endothelial cells (PMVECs) was examined after 24 h of treatment with 1 μM E2. Compared to controls, BMPR2 mRNA expression measured by quantitative PCR significantly decreased after 24 h of E2 in both normal human lymphocytes (*P *< 0.05) (Figure 3A) and PMVECs (*P *< 0.01) (Figure 3B) relative to the housekeeping gene HPRT.

To explore whether this response was unique to E2, expression of BMPR2 mRNA by PMVECs was examined after 24 h of treatment with E3 (estriol). Given the high levels of E3 during pregnancy, which is a time of particular vulnerability to PAH presentation, response to E3 was explored. Treatment with 10 nM (*P *= 0.001), 30 nM (*P *= 0.001), and 100 nM (*P *= 0.009) of E3 resulted in significantly lower BMPR2 gene expression compared to the unexposed control PMVECs.

Given the association of BMPR2-associated PAH with inappropriate cellular proliferation, we investigated the impact of stimulating with a known mitogen (PMA) on BMPR2 gene expression [32]. We found that PMA alone was sufficient to suppress BMPR2 expression to a degree comparable to E2 alone (Figure 3C). This finding was not surprising, given the known effect of PMA to increase expression by genes known to restrict cellular proliferation, and increase gene expression by genes known to promote cellular proliferation [32,33]. However, the combination of PMA and E2 caused a much more dramatic, roughly four-fold reduction in BMPR2 expression, which suggests enhanced reduction in BMPR2 gene expression in the setting of cellular proliferation.

### Reduction of BMPR2 gene expression by exposure to E2 occurs via ER binding to the BMPR2 promoter

**Figure 4:**
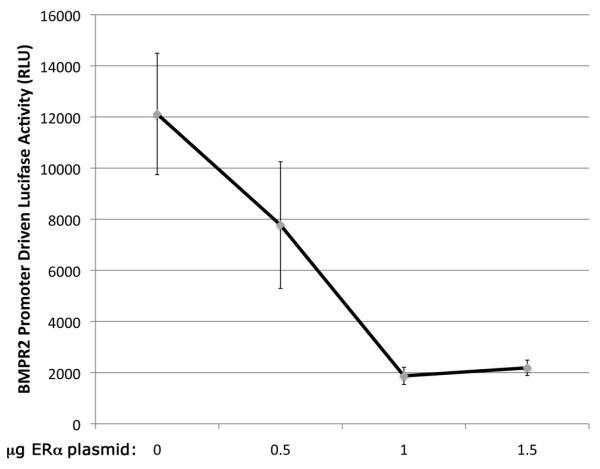
**In the presence of estrogen, addition of estrogen receptor to a cell line without endogenous expression reduces BMPR2 promoter-reporter activity, in a dose-response (*P *= 0.0013 by Pearson correlation)**.

Results in Figures 1 and 2 suggest that increasing levels of estrogen receptor ought to result in decreased BMPR2 expression. To test this directly, we used COS-7 cells, which lack endogenous estrogen receptors. COS-7 cells were transfected with noted amounts of ER expression plasmid and a constant amount of BMPR2 promoter-luciferase reporter plasmid. As expected, increasing levels of ERα plasmid resulted in decreasing levels of luciferase expression from the BMPR2 promoter driven reporter construct as normalized to tk-Renella (Figure 4).

### While E2 reduced BMPR2 gene expression, it appears to increase Smad-dependent BMP pathway activity

**Figure 5:**
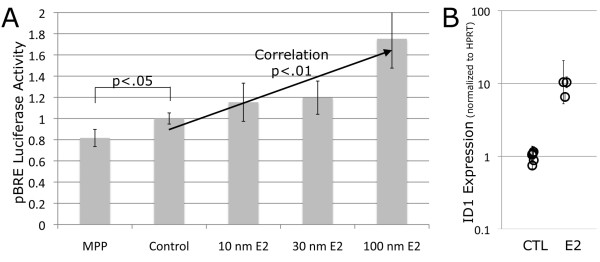
**While BMPR2 gene expression is reduced, canonical BMP pathway activity is increased by exposure to E2**. **(A) **A BMP responsive plasmid (pBRE system) studies: pBRE luciferase activity, a measure of BMP activity, is increased with exposure to increasing doses of E2. Uniform exposure to 50 ng of BMP4 in concert with exposure to MPP or varying doses of E2 occurred. Exposure to MPP, an ERα antagonist, resulted in reduced luciferase expression, while E2 exposure increased luciferase expression. **(B) **Consistent with this finding, gene expression of the canonical BMP signaling target (ID1) is increased with exposure to 10 nm E2 for 16 h.

To determine whether changes in BMPR2 transcript level brought about a functional change in Smad-dependent BMP pathway activity (the canonical BMP pathway), luciferase reporter assays were performed. pBRE, which measures BMP-specific Smad activity, and thus canonical signaling, was tested. We found that, in contrast to BMPR2 gene expression data, 16 h of E2 exposure at varying concentrations increased canonical BMP pathway signaling as measured by pBRE activity. This experiment established that while BMPR2 gene expression is reduced, the canonical BMP signaling pathway activity is not dampened in response to increasing doses of E2 *in vitro*. However, administration of the ERα antagonist methyl-piperidino-pyrazole (MPP) reduced canonical BMP activity below control. As pBRE represents a synthetic assay, we examined expression of the Smad-dependent canonical BMP transcriptional target ID1. ID1 transcript expression was elevated 10-fold following 16 h of exposure to 10 nm of E2 (Figure 5).

## Discussion

In this study, we have shown that BMPR2 has approximately 25% lower expression in lymphocytes cultured from female humans compared with males (Figure 1A), and approximately 15% lower expression in whole lung from ovariectomized female mice as compared to males (Figure 1B). In both of these cases, the actual estrogen exposure was the same for male and female groups, but the level of estrogen receptor was increased in females (Figure 1C). This led us to believe that estrogen effect on BMPR2 expression was through canonical estrogen receptors. There was an evolutionarily conserved estrogen receptor binding site in the BMPR2 promoter; we confirmed its functionality by gel-shift assay and its strength by its ability to out-compete the canonical estrogen receptor target vitellogenin (Figure 2). We found that direct administration of estrogen suppressed BMPR2 signal by 15% in cultured lymphocytes and in pulmonary microvascular smooth endothelial cells, but that in the presence of a proliferative signal this suppression could increase to 80% (Figure 3). We then used a cell line missing the estrogen receptor, and showed that we could add it back to get a six-fold reduction in BMPR2 expression in a dose-responsive manner (Figure 4). However, the reduction in BMPR2 gene expression at baseline was not reflected in a reduction in Smad-dependent effects including ID1; non-canonical BMPR2 signaling was not assessed (Figure 5). In total, these data indicate that under normal circumstances, increased estrogen or increased estrogen receptor decreases BMPR2 expression by approximately 15%, but under extreme circumstances (for example, proliferation or complete lack of estrogen receptor) the total potential effect is a five- to six-fold change. If this reduction is relevant to PAH pathogenesis, the mechanism may involve downstream signaling by BMPR2 that is not along the canonical BMPR2 signaling cascade.

These results are relevant to the disease we study, pulmonary arterial hypertension, both because of the high female prevalence, but also because we have shown that among those with a BMPR2 mutation, it is women who metabolize estrogen into the relatively more estrogenically active 16α-hydroxyestrone compound that develop disease [15-17]. In contrast, women who metabolize estrogen into 2-hydroxyestradiol or 2-hydroxyestrone are less likely to develop PAH [19]. Several of the enzymes responsible for estrogen metabolism, including CYP1B1, have been studied and are expressed at the mRNA and protein levels in lung tissue; not surprisingly, there is heterogeneity of expression among individuals [34]. It has been demonstrated that human bronchial epithelial cells metabolize estrogen *in vitro *to hydroxyestrogens such as 2-hydroxyestrone and 16α-hydroxyestrone; however, while biologically plausible, studies in pulmonary microvascular endothelial cells are lacking [35].

Since in most studies estrogen is protective of the pulmonary vasculature, the increased prevalence among women might be best explained by the simple hypothesis that gender differences occur through suppression of BMPR2. On a broader level, these results, while novel, might be expected. Whether or not the changes in BMPR2 expression we detected are specific to estrogens or also seen with other hormones, such as thyroid hormone and glucocorticoids requires further investigation. Thyroid abnormalities are well documented among PAH patients, and there is experimental data to suggest that glucocorticoids potentiate cellular proliferation among the vascular smooth muscle cells derived from idiopathic PAH patients [36]. Insulin resistance and perhaps the metabolic syndrome appear more prevalent among PAH patients, as well [37].

While this study evaluates BMPR2 expression at the molecular level, the PAH phenotype and the evolution of clinical disease among PAH patients is quite complicated. Data now exist to suggest that while PAH is more prevalent among females, incidence rates may not fully explain the gender discrepancy. For example, females have better right ventricular contractility compared to males at the time of diagnosis, which may contribute to a potential survival advantage [38,39]. Females with PAH also may respond better to certain therapies, as was recently shown to be the case in a pooled analysis of subjects treated with endothelin receptor antagonists [40]. Ultimately, there are two critical issues for human subjects at risk: (1) do you become a patient (develop PAH); and, (2) how long will you survive with PAH. Determining the diverse array of pieces that contribute to the 'gender puzzle' in PAH should ultimately contribute to better therapy for all PAH patients, and perhaps disease prevention. Estrogen-associated modification of BMPR2 expression may be one piece of that puzzle, although much work remains to be done.

This study has several limitations. For example, the human lymphocyte samples used were immortalized, so that they were equal in hormone exposure; this allowed for milieu uniformity but may not accurately replicate the actual circulating hormone exposure in human subjects. Also, we were unable to distinguish between ERα and ERβ effect, although we understand that in many cases the effect of the two is indistinguishable. Also, since our previous work implicated specific products of estrogen metabolism as a risk factor in PAH, comprehensive studies matching specific estrogen metabolites to specific receptors in the context of suppression of BMPR2 expression are warranted PAH [19,41].

Furthermore, while we detected a reduction in BMPR2 gene expression, the measurable functional consequences on BMP signaling in the unstimulated state did not suggest reduced Smad-dependent (canonical BMP signaling) effects. However, Smad-independent effects were not measured. We suspect that under normal circumstances the influence of estrogens upon BMPR2 is not relevant, and only becomes meaningful in the context of injury repair, when BMPR2 appears to be in disequilibrium with the canonical TGFβ signaling pathway [42,43]. As with BMPR2, estradiol decreases expression of TGFβ1 and the type 1 TGFβ receptor in the lung vasculature [44]. Further investigations are needed, but our findings may reflect the complicated balance of BMPR2, the TGFβ pathway and associated events that result in PAH among susceptible subjects (such as those with low BMPR2 at baseline), or the possibility that Smad-independent effects mediate downstream ramifications of BMPR2 gene expression changes. In the context of cellular injury, a delicate balance of cellular differentiation and renewal characterize the normal healing process, and it is possible that enhanced estrogen exposures disrupt this balance. The fact that estrogen exposure alters BMPR2 expression and downstream signaling, while clearly complex, is novel and may be relevant to PAH pathogenesis. Further studies will be required to separate these related findings.

## Conclusions

These studies strongly support the hypothesis that estrogen suppresses BMPR2 expression through direct BMPR2 promoter binding by the estrogen receptor. This provides one logical explanation for our finding of reduced BMPR2 gene expression among females compared to males, and may contribute to the significant gender disparity in pulmonary arterial hypertension. In addition, this has potential implications for estrogen-BMP cross-talk in many systems, while providing another example of variation in gene expression according to gender.

## Competing interests

The authors declare that they have no competing interests.

## Authors' contributions

EA provided scientific design, wrote the manuscript, performed data management, performed statistical analyses, and participated in the clinical field and laboratory work. RH performed study design, molecular biology laboratory analyses related to gene expression, and scientific review. AR performed laboratory analyses and scientific review. JP and JL had primary responsibility for scientific design, scientific oversight of this and related projects, and manuscript review. CY participated in study design and statistical analysis oversight. TB, EG, RG, and SG all contributed to the laboratory analyses presented, with TB participating in northern blotting and luciferase reporter assays, EG responsible for PMVEC culture work and exposures and real-time PCR measures, RG and SG participating in cell culture work, plasmid construction, and animal model studies. JW and KL contributed to project scientific oversight and manuscript writing, and JW directed animal study work while KL directed the luciferase EMSA, blotting, and luciferase assays. JP and JL participated in cell culture work and cohort maintenance, scientific design, and manuscript review. All authors read and approved the final manuscript.
